# Supplementary material for: High-performance photonic transformers for DC voltage conversion
Source: Nat Commun. 2021 Aug 3;12:4684. doi: 10.1038/s41467-021-24955-3 (PMC8333049; doi:10.1038/s41467-021-24955-3)
Supplement: Supplementary file 1 — Supplementary Information for High-Performance Photonic Transformers for DC Voltage Conversion [file 41467_2021_24955_MOESM1_ESM.pdf]

Supplementary Information for

High-Performance Photonic Transformers for DC Voltage Conversion

Bo Zhao[1,3,¶], Sid Assawaworrarit[1,3], Parthiban Santhanam[1], Meir Orenstein[1,2], and Shanhui Fan[1,*]

[1]Department of Electrical Engineering, Stanford University, Stanford, California, USA

[2]Department of Electrical Engineering, Technion Israel Institute of Technology, Haifa, Israel

[3]These authors contributed equally.

[¶]Current address: Department of Mechanical Engineering, University of Houston, Houston, Texas, USA

* *To whom correspondence should be addressed. Email: shanhui@stanford.edu.*

	LED	PV
active region material	GaAs	Si
bandgap	1.424 eV	1.12 eV
SRH lifetime (τ)	28.46 ns	0.551 μs
radiative recombination coefficient (B)	$1.12\times10^{-10}\text{cm}^3$ / s	$1\times10^{-14}\text{cm}^3/\text{s}$
Auger coefficient (C)	$4.6\times10^{-30}\ \text{cm}^6/\text{s}$	$1.3\times10^{-30}\ \text{cm}^6/\text{s}$
active region area (A)	$0.2\ \text{mm}\times 0.2\ \text{mm}$	$2.65\ \text{mm}\times 2.65\ \text{mm}$
active region thickness (t)	44.3 nm	10.24 μm
series resistance (R_s)	$1.47\ \text{m}\Omega\cdot\text{cm}^2$<br>$1.43\ \text{m}\Omega\cdot\text{cm}^2$	$2.18\ \Omega\cdot\text{cm}^2$
shunt resistance (R_{sh})	$5.4\ \Omega\cdot\text{cm}^2$	$1.49\ \text{k}\Omega\cdot\text{cm}^2$
LED light extraction efficiency (η_{EXT})/PV EQE (η_{RES})	0.39	0.897
doping level	intrinsic	$\text{N}_\text{d} = 2.52\times10^{15}\ \text{cm}^{-3}$
view factor $f_{\text{LED->PV}}$	0.73	

Supplementary Table 1. **Parameters of the LED and the PV cell from measurement and fitting the experimental data.** The active region area and the sensitivity of the PV cell are provided by the product datasheets. The bandgaps are obtained from Supplementary Reference 1. We note that the radiative recombination coefficient and the Auger coefficient for PV cells are directly taken from Supplementary Reference 1 since they have negligible effects on the performance of the device. The series resistances include resistances at both board level and package level, as well as resistances within the semiconductors.

	LED	PV
SRH lifetime (τ)	16.7 μs	35 ms
radiative recombination coefficient (*B*)	$7\times10^{-10}\,\text{cm}^3/\text{s}$	same as in Table 1
Auger coefficient (*C*)	$3.5\times10^{-30}\ \text{cm}^6/\text{s}$	same as in Table 1
series resistance (R_s)	same as in Table 1	$0.32\ \Omega\cdot\text{cm}^2$
LED light extraction efficiency (η_{EXT})/PV EQE (η_{RES})	0.8	same as in Table 1
view factor $f_{\text{LED->PV}}$	1	

Supplementary Table 2. **Parameters of the LED and the PV cell used in improvements shown in Figure 4d.** The PV parameters are obtained from Supplementary Reference 2. The LED parameters are from Supplementary Reference 3, with the light extraction efficiency obtained from the product sheet of a high-efficiency infrared 850 nm LED (Osram LZ1-00R402).

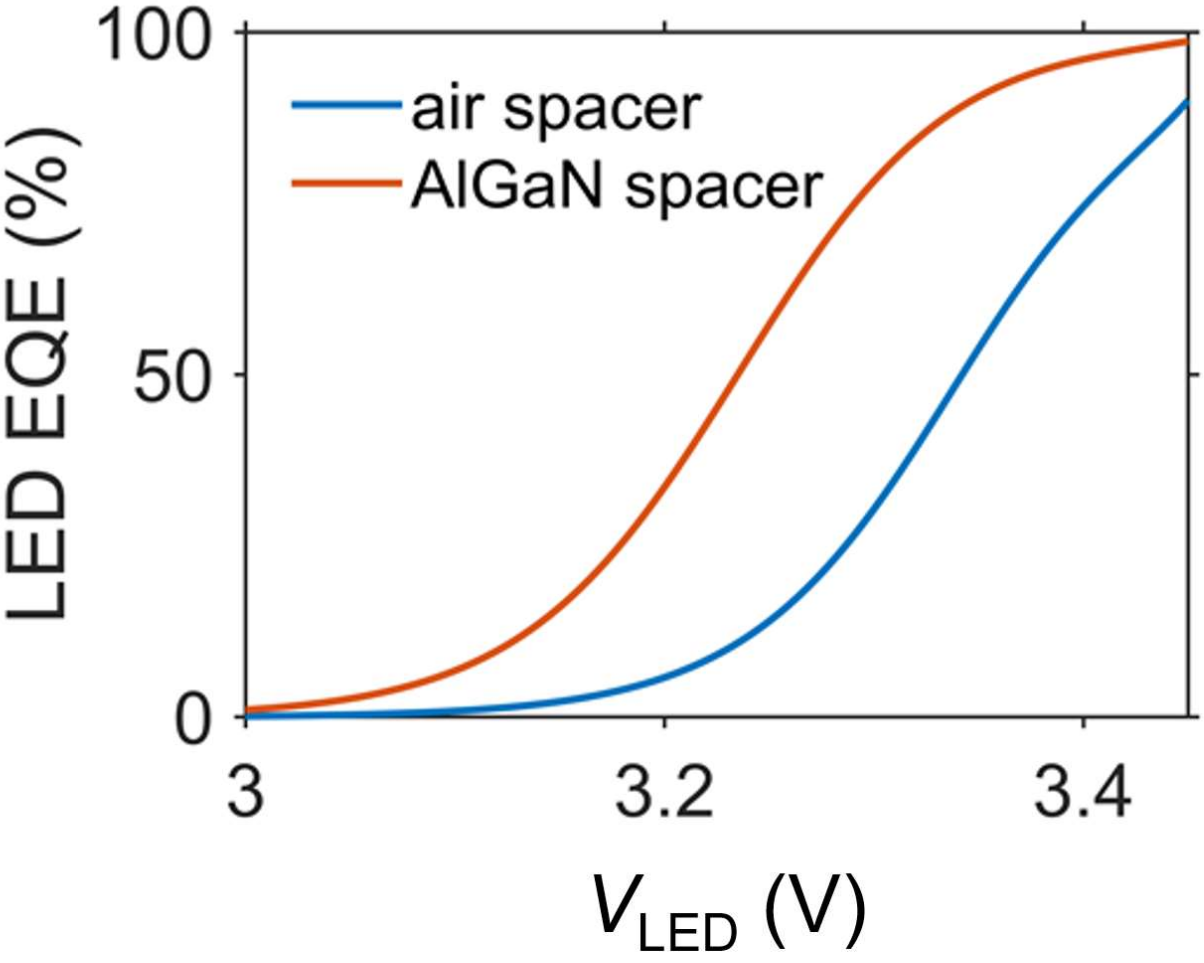

Supplementary Figure 1. **External quantum efficiency (EQE) of the GaN LED for the air spacer and AlGaN spacer cases.** In computing the curves, the PV cell is assumed to be in short circuit condition.

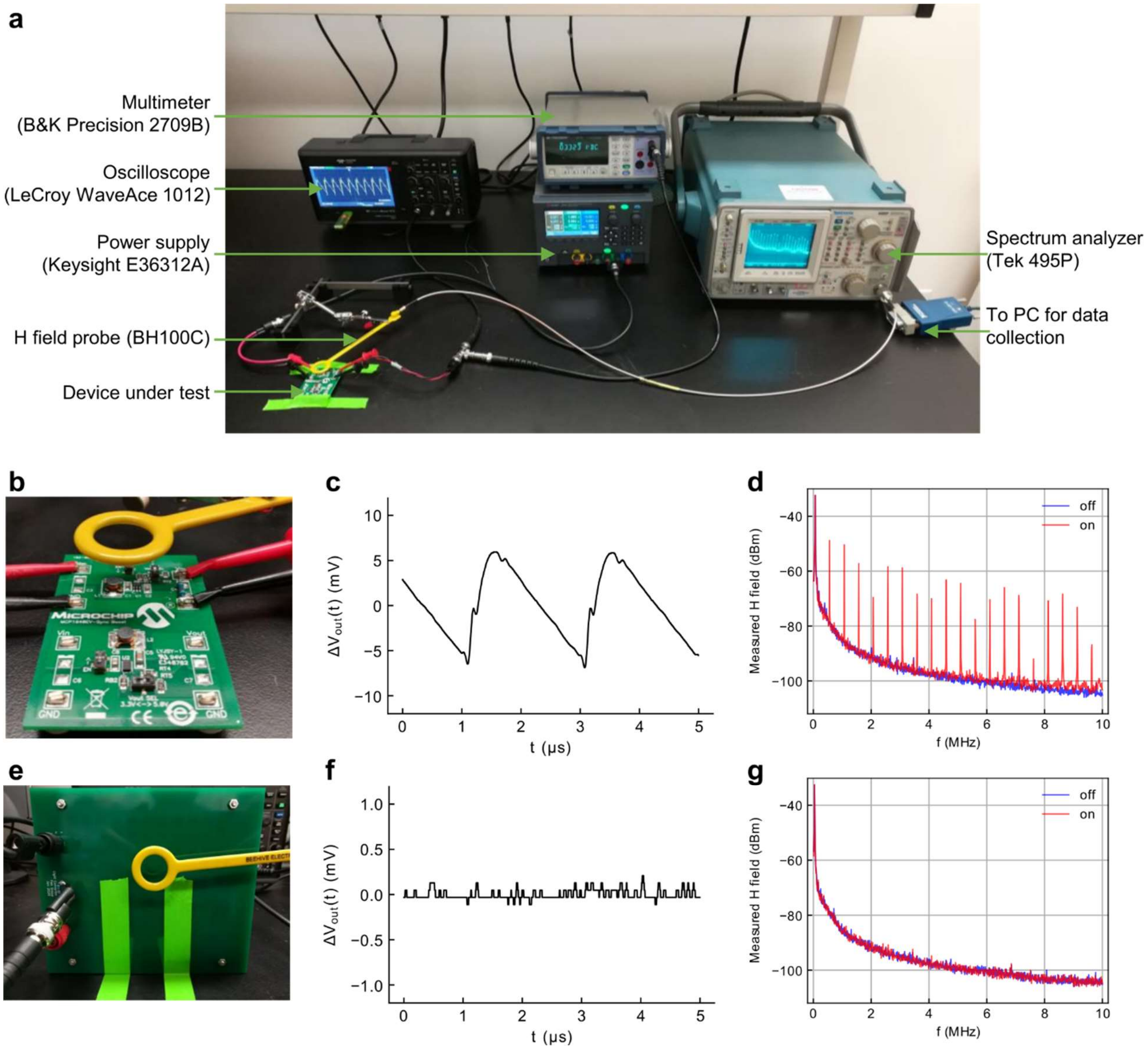

Supplementary Figure 2. **Measurement setup and results for output voltage fluctuations and electromagnetic field emissions of commercial switch mode converter and photonic transformer. a**, Measurement setup. **b**,**e**, Close-up photos of the circuits under test: switching converter (b) and photonic transformer circuit (e). **c**,**f**, Output voltage waveforms for the switching converter (c) and the photonic transformer (f). **d**,**g**, Power spectra of the electromagnetic field emitted from the switching converter (d) and the photonic transformer (g) measured when the corresponding circuit is powered on (red) and off (blue).

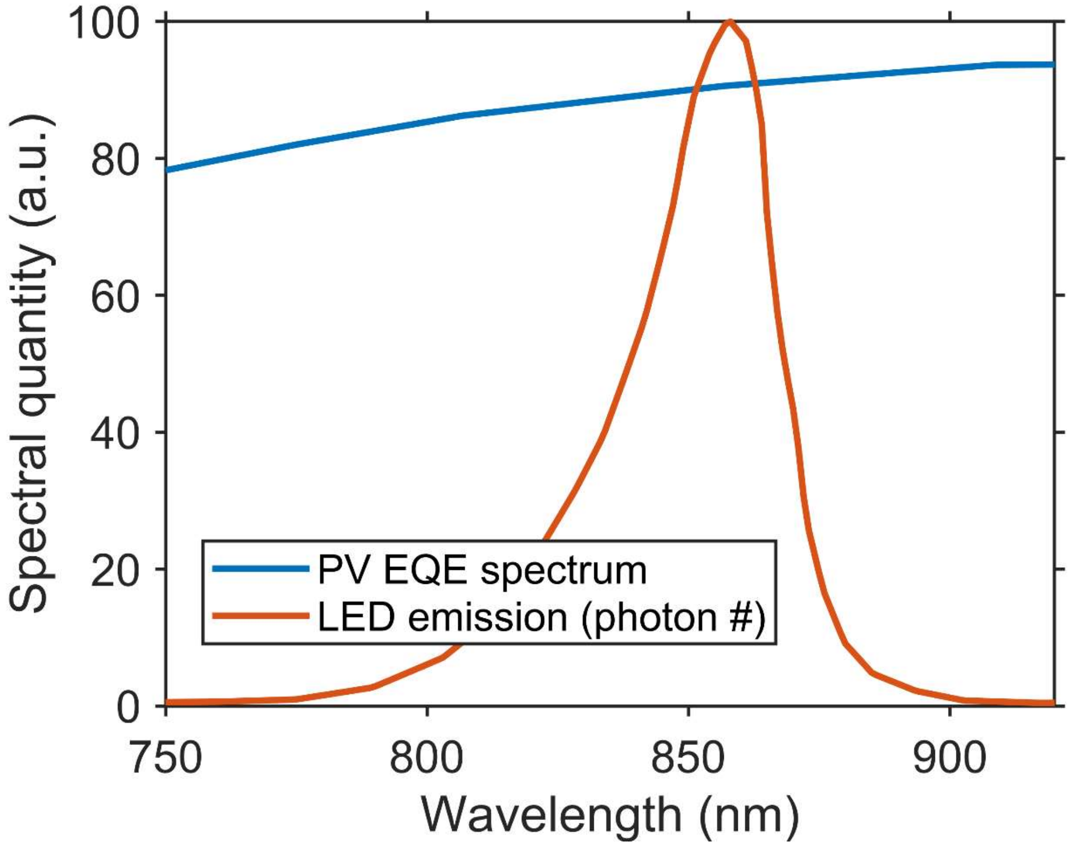

Supplementary Figure 3. **The spectrum of photon flux from the electroluminescence of the GaAs LED and the external quantum efficiency spectrum of the Si PV cell.** Obtained from the datasheets of the devices used in our experiments.

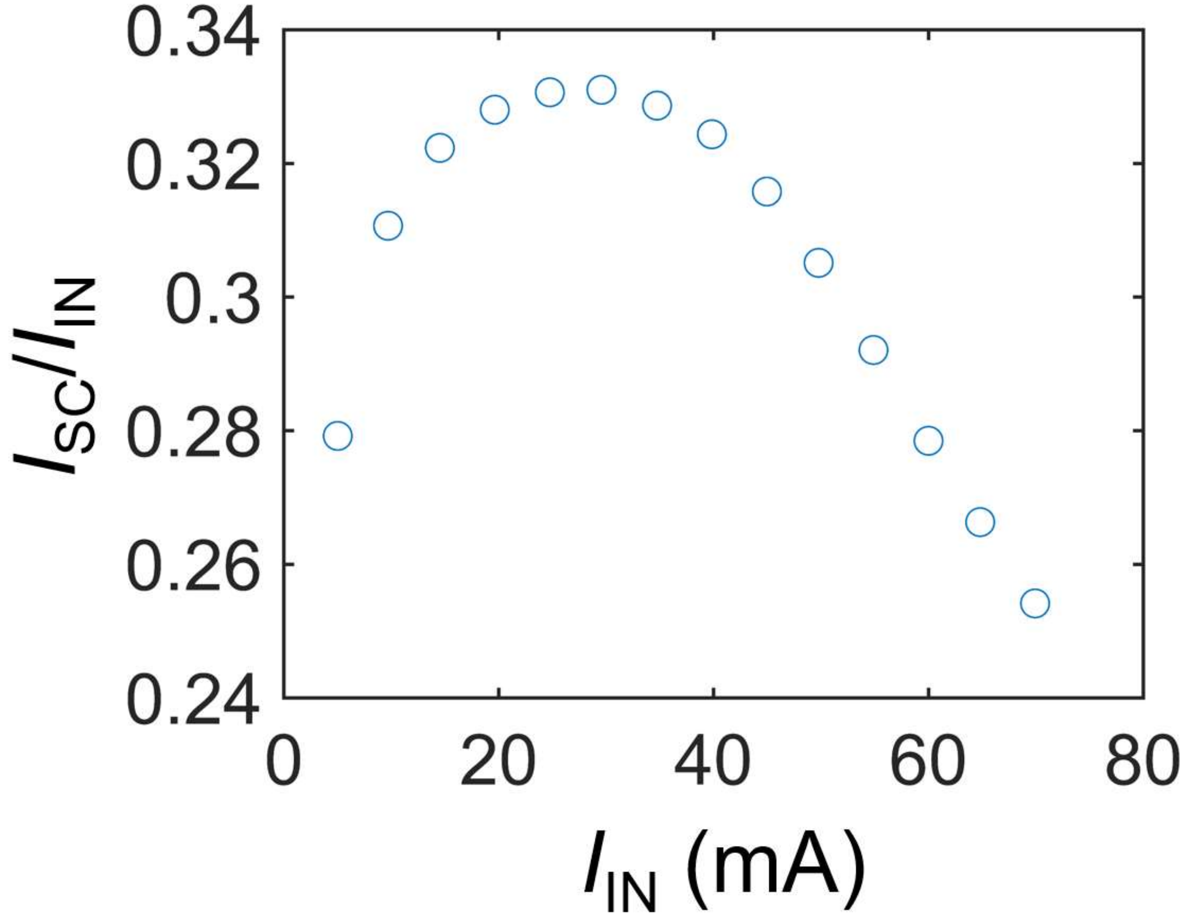

Supplementary Figure 4. **Measurement of a device consisting of one PV cell facing one LED**. Plotted here is the ratio of the short circuit current of one PV cell to the input current of one LED, as a function of the input current of the LED.

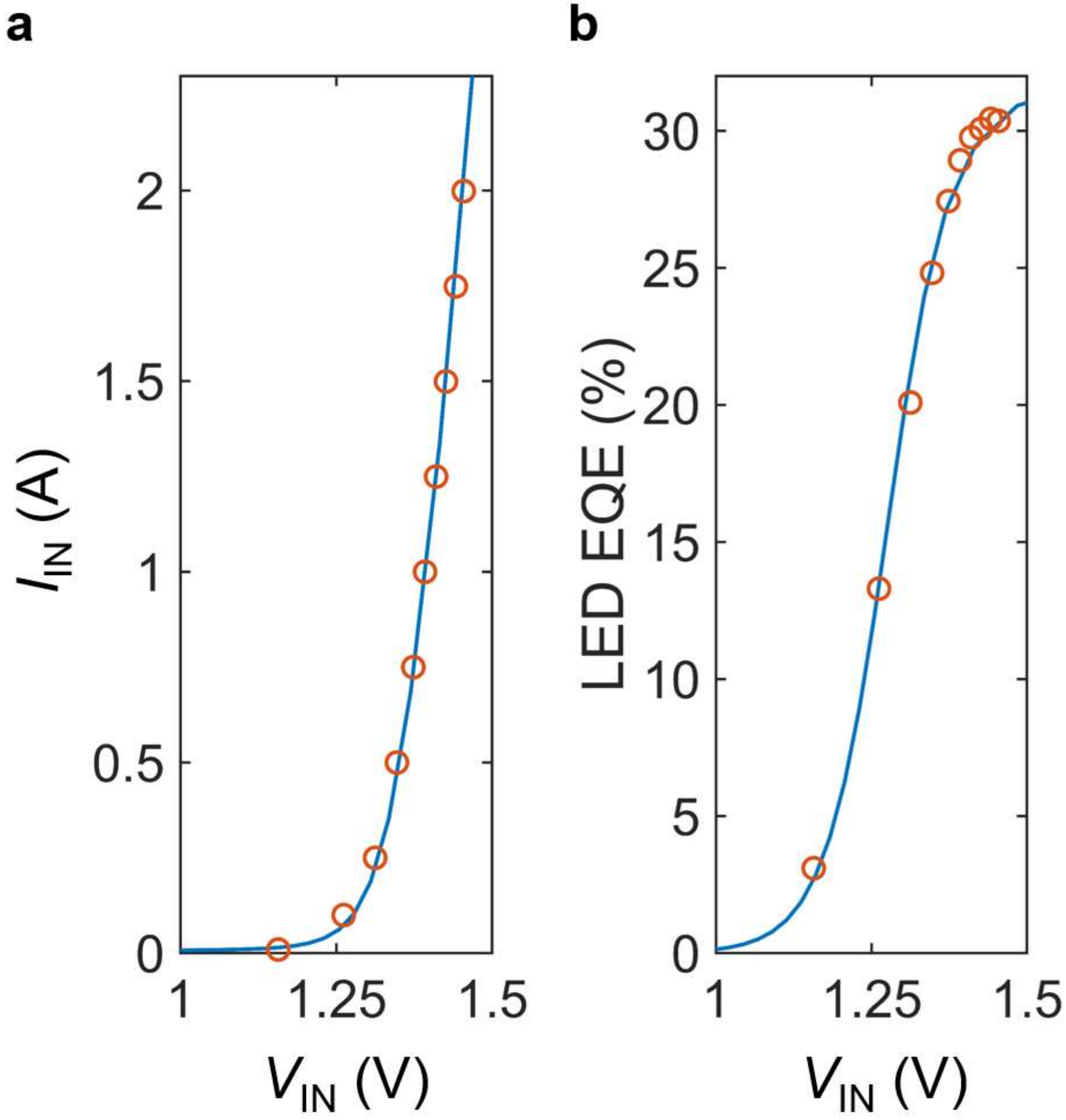

Supplementary Figure 5. **Measured (round dots) and fitted *I-V* curve and external quantum efficiency (EQE) curve of the LED board.** The parameters obtained from numerical fitting are listed in **Supplementary Table 1.**

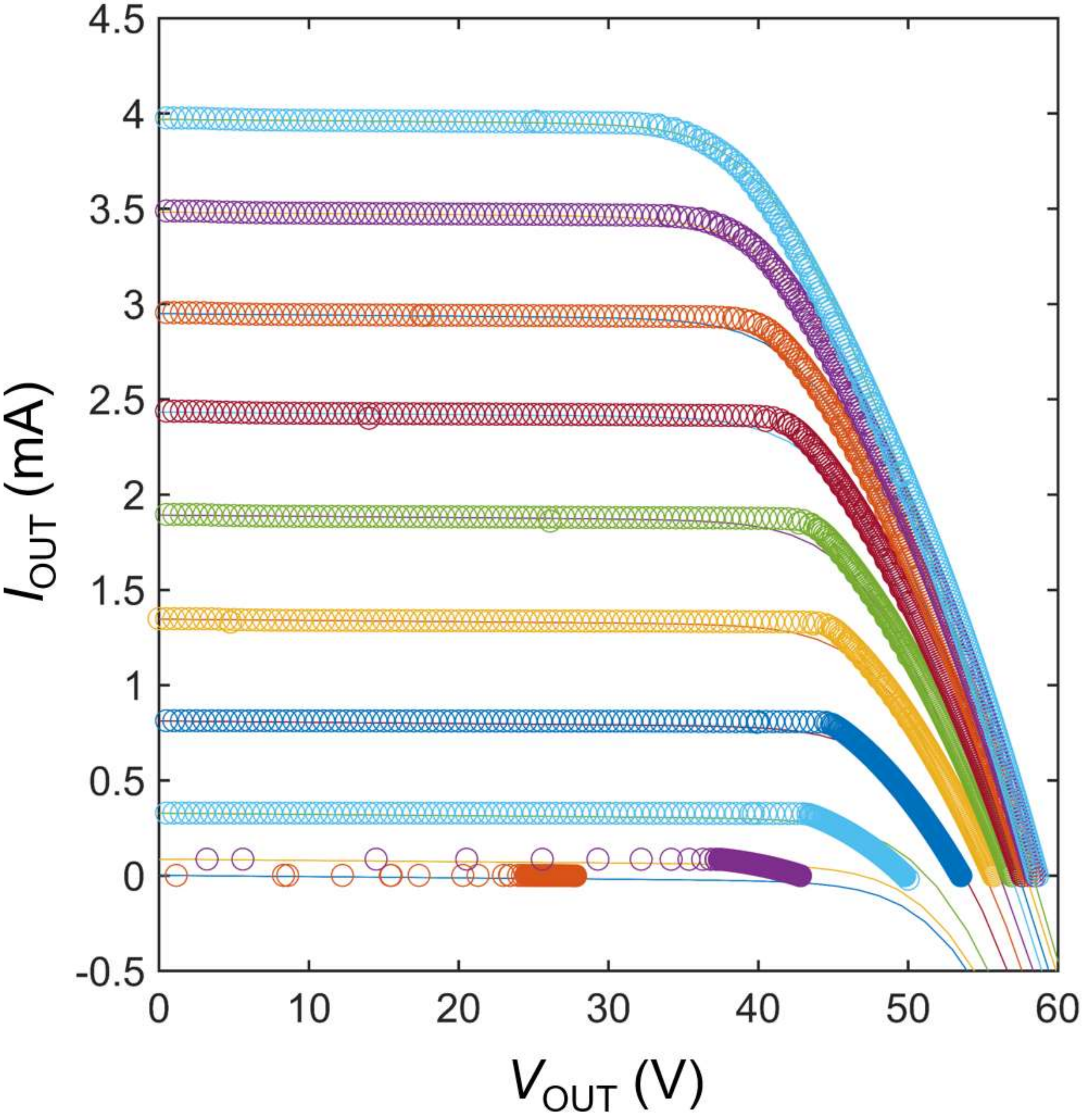

Supplementary Figure 6. **Measured (round dots) and fitted *I-V* curves (continuous lines) of the PV cell array at different input power levels.** The parameters obtained from numerical fitting are listed in **Supplementary Table 1.**

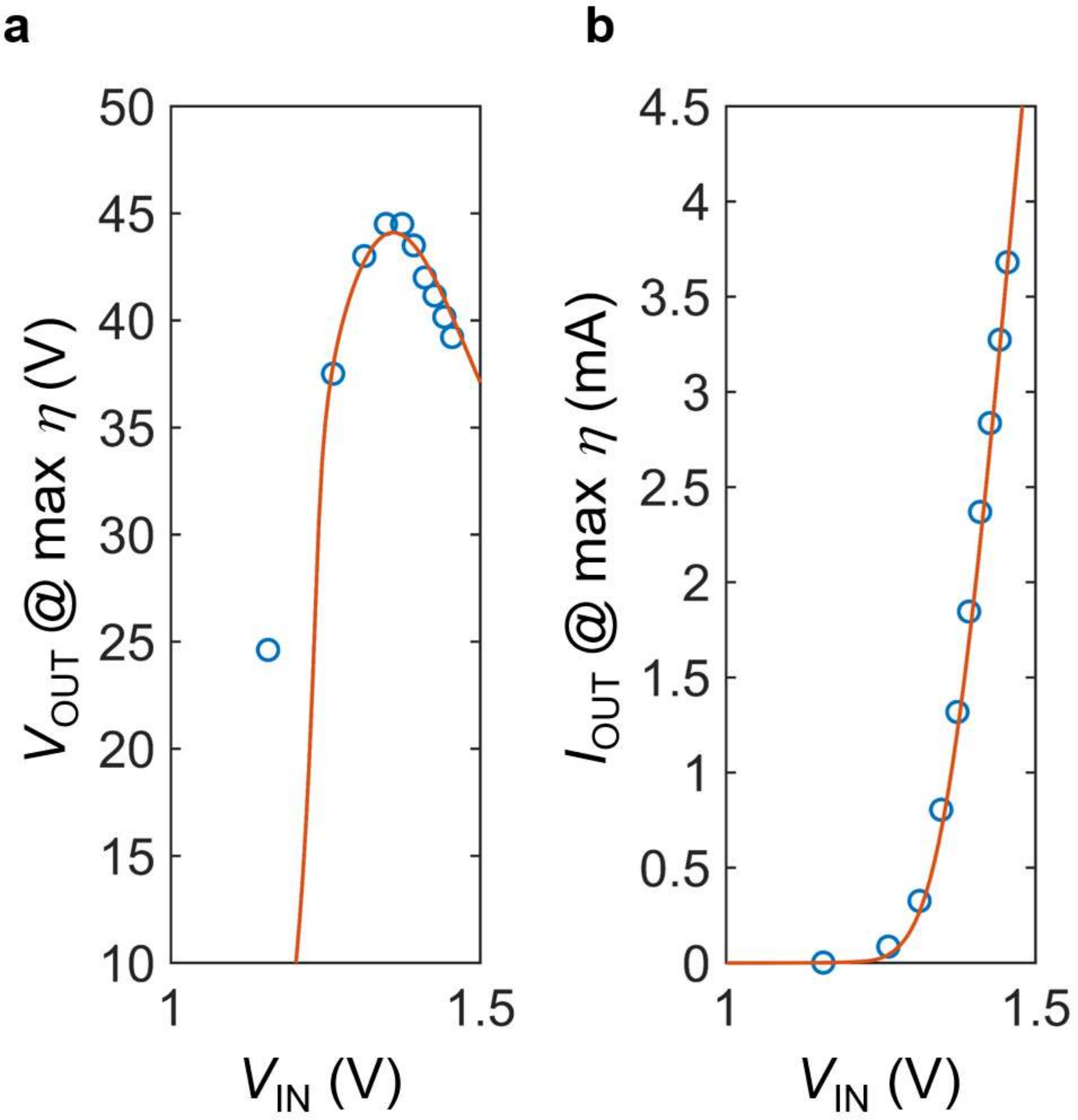

Supplementary Figure 7. **Measured (round dots) and model predictions (continuous lines) of the output voltage and current at the maximum efficiency point.**

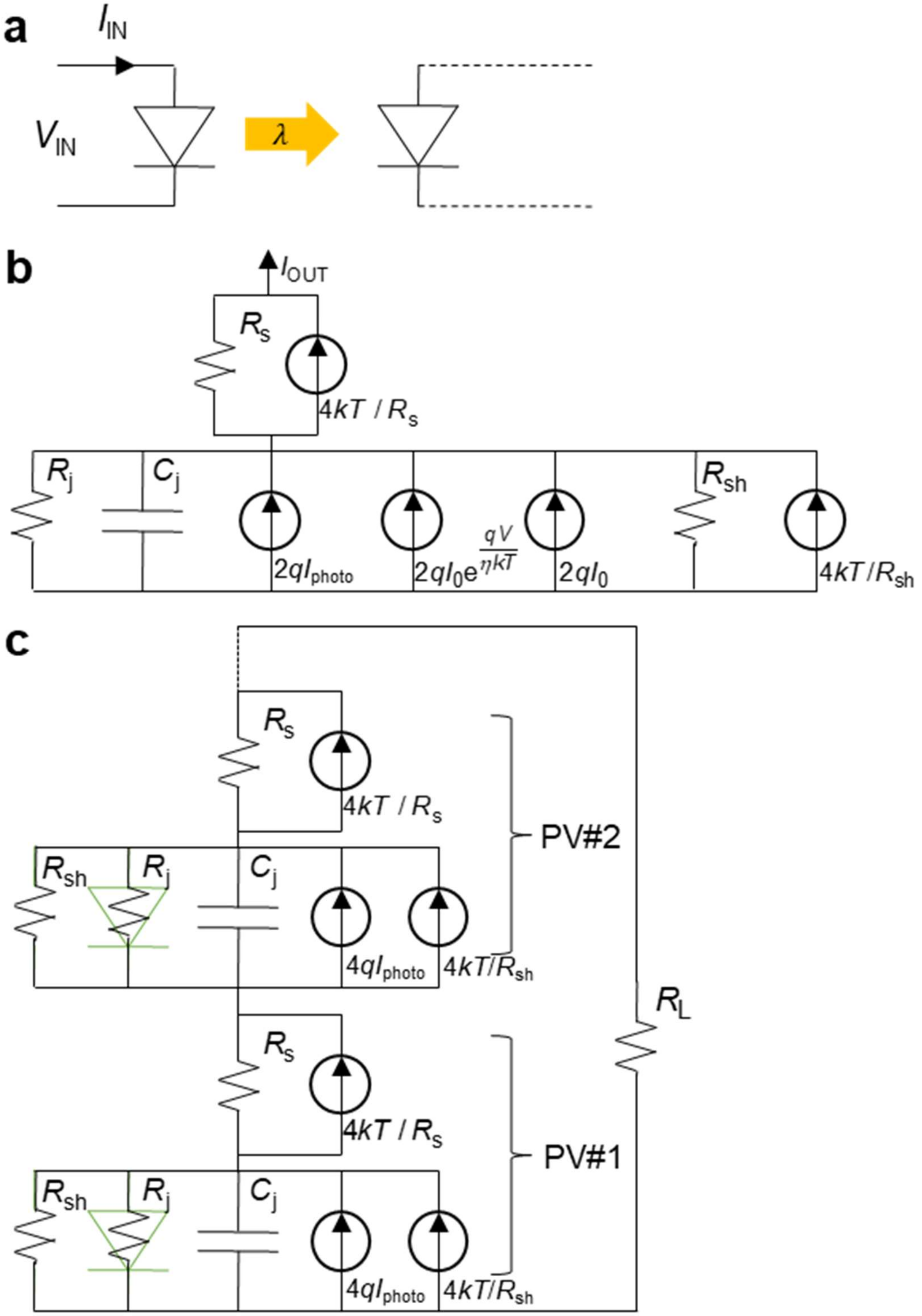

Supplementary Figure 8. **Noise analysis for the photonic transformer circuit.** **a**, A circuit consisting of one LED and one PV cell. **b**, The noise model for the circuit shown in a. **c**, Noise model for a photonic transformer circuit containing N LEDs and N PV cells.

Supplementary References

1. I. P.-t. Institute, NSM archive - physical properties of semiconductors; *The Ioffe Physical-Technical Institute of the Russian Academy of Sciences* (1998).
2. Yoshikawa, K. et al. Silicon heterojunction solar cell with interdigitated back contacts for a photoconversion efficiency over 26%. *Nat. Energy* **2**, 17032 (2017).
3. Chen, K. et al. High-performance near-field electroluminescent refrigeration device consisting of a GaAs light emitting diode and a Si photovoltaic cell. *J. Appl. Phys.* **122**, 143104 (2017).
